# Eosinophilic Gastroenteritis with a Relapsing and Remitting Course with Presence of Autoimmune Antibodies

**DOI:** 10.1155/2020/1745834

**Published:** 2020-08-18

**Authors:** Chathuranga Lakmal Fonseka, Sunali Nanayakkara, S. D. A. L. Singhapura, H. M. M. Herath, C. K. Bodinayake

**Affiliations:** ^1^University Medical Unit, Teaching Hospital Karapitiya, Galle, Sri Lanka; ^2^Faculty of Medicine, University of Ruhuna, Matara, Sri Lanka

## Abstract

**Background:**

Eosinophilic gastroenteritis (EGE) is an uncommon disease characterized by eosinophilic infiltration of the digestive tract, which occurs due to an uncertain aetiology. Although autoimmune diseases can later present as EGE, it is unusual for EGE to have positive autoimmune antibodies without the presence of an overt autoimmune disease. *Case presentation.* We report a 38-year-old previously healthy man who presented with abdominal discomfort and loose stools with pleural and peritoneal effusions progressing over several weeks. His investigations revealed severe eosinophilia in peripheral blood and ascitic fluid, and a laparoscopic full-thickness biopsy from the ileum demonstrated infiltration of eosinophils in all three layers of the intestine. There were no clinical features or investigations suggestive of parasitic disease, other diseases associated with eosinophilia, or autoimmune disease. Further investigations showed a highly positive ANA, positive p-ANCA, but did not meet the criteria to diagnose a specific autoimmune disease. The eosinophilia responded to an elimination diet with gradual resolution of eosinophilia and effusions, and once it reappeared after introduction of a normal diet.

**Conclusion:**

EGE presenting as peripheral blood and ascitic fluid eosinophilia with the presence of pleural and/or peritoneal effusions is uncommon. Eosinophilic gastroenteritis can be associated with autoantibody positivity without any evidence of overt autoimmune disease manifestations. Elimination diet can be used as a potential option to prevent recurrences of EGE.

## 1. Background

Eosinophilic gastroenteritis (EGE) is a rare inflammatory disease characterized by eosinophilic infiltration of the digestive tract [1], with an approximate prevalence ranging from 1 in 100,000 to 28 in 100,000 population in different studies [2, 3]. Up to 50% of patients having EGE can have a personal or family history of atopic disease, including asthma, rhinitis, eczema, and drug or food intolerance [1, 4]. Emerging evidence for the pathogenesis of EGE points towards immunoglobulin E-mediated allergy and delayed T-helper type 2 response triggered by a variety of stimuli [4]. Additionally, interleukins 3–5 and tumor necrosis factor-alpha are involved in eosinophil recruitment, homing, and prolonging eosinophilic action in the intestinal wall and are considered potential targets for molecular therapy [5].

EGE is classified into three types on the basis of the depth of eosinophilic infiltration [6]. They are mucosal, muscular, and serosal. While the mucosal variety is the most common, the serosal variety remains the rarest. Clinical features vary depending on the location, extent, and depth of infiltration of the gastrointestinal wall. Clinical presentation is usually heterogeneous. Reported clinical presentations include abdominal pain or discomfort, changes in bowel habits with increased or reduced frequency of bowel opening, mucous in stools, per rectal bleeding, tenesmus, intractable vomiting, and abdominal distension [1,3]. With serosal involvement, ascites can occur, and rarely it may present with features of intestinal obstruction [7]. Diagnosis of eosinophilic gastroenteritis requires three criteria to be met, which include clinical suspicion, histological evidence of eosinophilic infiltration of bowel, and exclusion of other pathological conditions which give rise to hypereosinophilia. To arrive at a histological diagnosis, a peak count of >15 intraepithelial eosinophils per high-power field is recommended as an absolute minimum number to arrive at a diagnosis [8]. Additional features that are not pathognomonic but may be helpful include eosinophil microabscesses, surface layering of eosinophils, basal layer hyperplasia, papillary lengthening, degranulating eosinophils, and lamina propria fibrosis and inflammation [8].

Dietary modification and oral steroids are the two main treatment modalities used in eosinophilic gastroenteritis [9]. Resolution of clinical symptoms, maintenance of histologic remission, and prevention of long-term complications such as remodeling and stricture formation of the bowel are the goals of therapy. Long-term steroid use raises the concern of common adverse reactions. Therefore, dietary therapy is considered an attractive treatment option. The available dietary approaches include an exclusive elemental diet with an amino acid-based complete liquid formulation, an allergy test-directed elimination diet, and an empiric elimination diet that excludes common disease-triggering foods. Still, there are no prospective studies comparing the efficacy of the different dietary approaches on EGE [9, 10].

EGE with a presence of autoimmune antibodies without any features of autoimmune disease is an unusual presentation not reported before in the literature. We report a rare presentation of a patient diagnosed to have eosinophilic gastroenteritis who had positive autoimmune markers and showed clinical and biochemical improvement with an elimination diet to maintain remission.

## 2. Case Presentation

We present a 38-year-old previously healthy male who admitted with progressive left-sided abdominal pain involving the periumbilical and epigastric region. Pain was dull with no radiation and progressed over a week. On the eighth day of the illness, he developed several episodes of watery stools and had no blood or mucus. Six months prior to this episode, he experienced a similar episode which lasted for two weeks and resolved spontaneously without any medication. On systemic inquiry, the patient did not have any history of arthritis, vasculitic rash, frothy urine, nasal polyps, a history of food allergy or atopy, asthma, or a history suggestive of neuropathy or vasculitis. He did not notice any adverse reaction to a specific food.

On examination, the patient was afebrile, not pale, and had difficulty in breathing. He was tachycardic with a pulse rate of 116 beats per min, and his blood pressure was normal. There were bilateral pleural effusions (more prominent on the right side) with shifting flank dullness in the abdomen. He did not have lymphadenopathy, hepatosplenomegaly, or ankle oedema. Initial blood counts showed marked leukocytosis with predominantly eosinophils (white cell count 14,200 × 10^3^/microliter, eosinophil count 5,580 × 10^3^/microliter). Blood picture showed severe eosinophilia with normal cellular morphology. The stool full report was negative for amoeba, ova, or cysts. Stool culture was negative. Liver function tests including albumin and globulin were normal and so were the inflammatory markers (ESR 14 mm/1^st^ hour, CRP 27 mg/L (normal less than 8)). Renal function and urine analysis were normal. Abdominal ultrasound confirmed the presence of significant pleural effusion and ascites. Ascitic fluid was aspirated, and the full report revealed the presence of protein 5.4 g/dl and white blood cells of 420 per cumm of which 95% were eosinophils. The serum IgE level was 774.2 IU/ml (normal range for adults <100 IU/ml). Upper gastrointestinal endoscopy and histology from serial biopsies from the oesophagus were normal.

**Figure 1:**
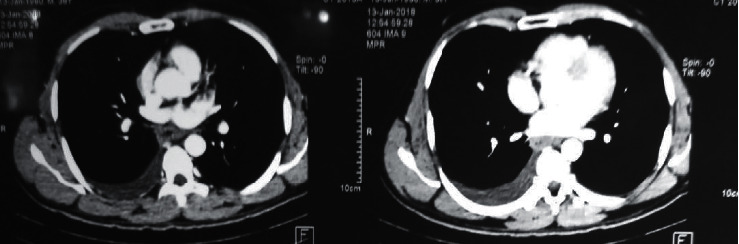
Contrast-enhanced computerized tomography of the chest showing right-sided pleural effusion.

**Figure 2:**
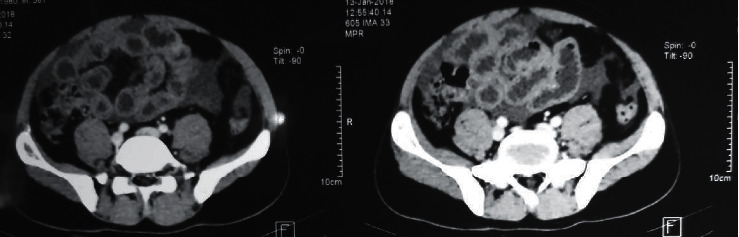
Contrast-enhanced computerized tomography of the abdomen showing thickened mucosa of the small intestine.

Since the ascitic fluid full report showed predominantly eosinophils with high protein concentration, contrast-enhanced CT of the abdomen and chest was ordered. It showed moderate pleural effusion on the right side (Figure 1) with gross ascites within the peritoneal cavity. Mucosal thickening of the wall of the gastric antrum, duodenum, and small bowel (Figure 2) was noted and suggested to investigate for the presence of gastric lymphoma or small bowel inflammation. However, mediastinal or visceral lymphadenopathy was not observed on imaging. Thereafter, we arranged a laparoscopic-guided assessment and a full-thickness biopsy from the ileum. Although no macroscopic abnormalities were detected, histology revealed moderate inflammation in the lamina propria, which is comprised of a significant number of eosinophils, lymphocytes, and a few plasma cells. Inflammation extended into the submucosa into the muscularis propria, which showed many intramural eosinophils (>20/hpf). The overall appearance favored the diagnosis of eosinophilic serositis.

His antinuclear antibody titer (an indirect immunofluorescent method) was high (1 : 1280), anti-double stranded (ds) DNA antibody was negative, and the rheumatoid factor was 64 IU/ml (normal range less than 8 IU/ml). Perinuclear anti-neutrophil cytoplasmic antibody (p-ANCA/MPO was positive, whereas cytoplasmic anti-neutrophil cytoplasmic antibody (c-ANCA/PR3) was negative. Both tests were carried out by the indirect immunofluorescent method and confirmed by enzyme-linked immunosorbent assay.

Initially, the patient was treated with a course of antiworm treatment and diethylcarbamazine, with poor clinical and haematological response. The eosinophil count rose up to 11,000 × 10^3^/microliter. After arriving at the diagnosis of eosinophilic gastroenteritis/serositis, the patient was initiated on an elimination diet with clinical resolution of symptoms and haematological improvement after a week. Therefore, we did not initiate him on oral steroids. In this particular diet plan, the patient was asked to abstain from six types of food which are considered probable food allergens including milk and dairy products, egg, soy, tree nut or peanut, and sea food (fish/shellfish) [11]. During the follow-up at six weeks, the absolute eosinophil count dropped to less than 500 × 10^3^/microliter. In a later time, when we did a stepwise reintroduction of individual food items, and we observed that after several weeks of introduction of fish, his eosinophil count rose to 1000 × 10^3^/microliter. After withdrawal of fish, the eosinophil count reduced to 300 × 10^3^/microliter after several weeks. Thereafter, he was advised to avoid fish and he did not have any symptoms to suggest a clinical recurrence. He was followed up at 6, 12, and 24 months after discharge with no recurrence of symptoms, eosinophilia, or features of an autoimmune disease. During the follow-up period, he continued to avoid fish but consumed other food.

## 3. Discussion

Eosinophilic gastroenteritis is an emerging disease which could present in many ways with respect to the severity and site of involvement. Our patient presented with a relapsing and a remitting course of vague complaints of abdominal pain and distension with loose stools, and the clinical and ultrasonic examination revealed pleural effusions and ascites. The main diagnostic clue was the presence of blood and ascitic fluid eosinophilia, which led us to perform an ileal full-thickness biopsy which showed abundant eosinophils. There was no evidence to suggest parasitic infection, and there was no convincing evidence to suggest autoimmune disease.

One of the major reasons that make this presentation unique is the positivity of markers of autoimmunity with a significant ANA titer, positive rheumatoid factor, positive p-ANCA, in the absence of autoimmune disease up to 2 years of follow-up. Though eosinophilic gastroenteritis has been diagnosed in a significant number of patients presenting with gastrointestinal symptoms, it has been described rarely to be associated with rheumatological conditions including rheumatoid arthritis, systemic lupus erythematosus, dermatomyositis, Sjogren's syndrome, overlap syndromes [12], polymyositis, and scleroderma [13, 14]. All these cases had clinical features of autoimmune disease during the time of diagnosis [13, 14]. Aslanidis et al. [12] described a case of EGE in a young female having systemic lupus erythematosus and limited scleroderma with positive antinuclear antibodies, antiribonucleoprotein (anti-RNP), anti-DNA and anti-*β*2-glycoprotein antibodies presenting with abdominal pain and ascites. In a systematic review, twenty cases of connective tissue disease associated with eosinophilic gastroenteritis were identified. Among them, the majority had systemic lupus erythematosus (35%) and rheumatoid arthritis (20%) [13]. Avgerinos et al. described a patient who had Churg-Strauss syndrome diagnosed five years back, presenting with chronic diarrhea for one-year duration. His upper and lower gastrointestinal tract biopsies showed eosinophilic infiltration of the lamina propria and muscularis mucosa consistent with eosinophilic gastroenteritis. The patient was treated with prednisolone and later with methotrexate [15].

All the above-described patients had met definitive diagnostic criteria of a connective tissue disorder, but our patient, though he had serological evidence, did not have any clinical manifestation even after 2 years of follow-up. In the literature, we did not come across any patient with eosinophilic gastroenteritis having positive autoimmune markers without fulfilling the diagnostic criteria of any autoimmune disease. Our patient did not have any evidence of autoimmune disease or did not meet the criteria to diagnose autoimmune disease associated with hypereosinophilia and yet had autoantibody screening positive for rheumatoid factor, ANA in a high titer, and p-ANCA. We think that he could be having an evolving connective tissue disorder such as eosinophilic granulomatosis with polyangiitis. Therefore, we suggest to perform autoimmune antibody screening in patients with EGE.

Our patient had similar episodes of abdominal pain and diarrhea in the past, but resolved without any intervention. Though this time he had disabling symptoms, it could have improved spontaneously or by implementation of dietary restrictions. The most commonly used treatment strategies include steroids and dietary treatment, which include an elemental diet or elimination diet. The elimination diet includes either of empiric removal of common trigger foods or allergic test-directed elimination [9]. Currently, most of the studies on the six-food elimination diet are conducted among patients with eosinophilic esophagitis [16]. As our patient had normal gastroscopic findings and histology, it was quite difficult to interpret his clinical course from the available literature. Dietary intervention, including the elemental diet and six-food elimination diet, could be effective measures to induce and maintain histological (and clinical) remission in patients with eosinophilic esophagitis [9]. A case report describes the use of a multiple-food elimination diet to induce disease remission in a 22-year-old woman with eosinophilic gastroenteritis. Initially, she was treated with oral steroids and had recurrence of symptoms on withdrawal of the drug. During dietary treatment, they identified dairy products and eggs as likely causative foods exacerbating symptoms, and thereafter, they could manage the patient without prednisolone [10]. Similarly, we attempted an elimination diet in our patient who had eosinophilic gastroenteritis. This could suggest that an allergic predisposing factor may have brought out a relapse at the presentation of the disease or there could be a link between autoimmunity and allergy. Steroid use can be a nonspecific immunosuppressant to treat autoimmune or allergic conditions with significant side effects on long-term usage. It has been described that mast cells, which play a key role in allergic reactions, can produce inflammatory mediators which can predispose the development of autoimmune processes. Activation of protein kinases by inflammatory cytokines and environmental stresses could contribute to both allergic and autoimmune diseases. This is further suggested by the presence of autoantibodies in some allergic conditions, elucidating an autoimmune basis for these conditions, as in our patient [17]. Furthermore, pathogenesis-related research is required to identify the triggers and pathways in view of developing a specific targeted therapy. However, we suggest to undertake an elimination diet as a plausible option to prevent recurrences of EGE and to maintain remission.

## 4. Conclusion

EGE presenting as peripheral blood and ascitic fluid eosinophilia with the presence of pleural and/or peritoneal effusions is uncommon. Eosinophilic gastroenteritis can be associated with autoantibody positivity without any evidence of overt autoimmune disease manifestation. An elimination diet can be used as a potential option to prevent recurrences in EGE. This could suggest an autoimmune allergic pathogenesis for EGE.
